# The Prevalence and Risk Factors of Hypokalemia in Pregnancy-Related Hospitalizations: A Nationwide Population Study

**DOI:** 10.1155/2021/9922245

**Published:** 2021-06-28

**Authors:** Chien-Wen Yang, Si Li, Yishan Dong

**Affiliations:** ^1^Renal Electrolyte and Hypertension Division, Hospital of the University of Pennsylvania, 3400 Civic Center Blvd, Philadelphia, PA 19104, USA; ^2^Department of Internal Medicine, Wright Center for Graduate Medical Education, 501 S Washington Ave, Scranton, PA 18505, USA; ^3^Department of Internal Medicine, Rochester General Hospital, 1425 Portland Ave, Rochester, NY 14621, USA

## Abstract

**Background:**

There are no nationwide population studies conducted to analyze the prevalence and risk factors associated with hypokalemia during pregnancy in the U.S.

**Method:**

We retrieved data from the Nationwide Inpatient Sample (NIS) and the National Inpatient Sample of Healthcare Cost and Utilization Project (HCUP) for pregnant patients with hypokalemia from 2012 to 2014. We used a chi-squared test to analyze categorical variables and an adjusted Wald test to compare quantitative variables. We applied logistic regression models to calculate adjusted odds ratios (ORs) with 95% confidence intervals (95% CIs) to identify the risk factors for hypokalemia. We used a *p* value <0.05 as the cutoff for statistical significance.

**Result:**

Among 12,431,909 pregnancy-related discharges, females of younger age (mean age 27.0 ± 6.2 vs. 28.1 ± 6.0, *p* < 0.001), of African American race, using government-paid insurance, with an income level in the first quartile, and of a higher Charlson Comorbidity Index score (≥1) were found to have a higher likelihood of hypokalemia during pregnancy (*p* < 0.001). Gestational hypertension (GH) (including pre-eclampsia and eclampsia, aOR 2.03, 95% CI 1.94–2.12, *p* < 0.001), hyperemesis gravidarum (aOR 33.18, 95% CI 31.61–34.83, *p* < 0.001), and post-partum hemorrhage (aOR 1.42, 95% CI 1.31–1.53, *p* < 0.001) were found to be independently associated with a higher rate of hypokalemia during pregnancy.

**Conclusion:**

The prevalence of hypokalemia during pregnancy was less than 1% in this large, nationwide population-based study. There were significant differences between those patients who developed hypokalemia during pregnancy. Notably, those who had hypokalemia were younger, of African American race, and of a low-income level. Congestive heart failure, coronary artery disease, Cushing's syndrome, GH, and hyperemesis gravidarum were found to be associated with hypokalemia during pregnancy.

## 1. Introduction

Hypokalemia is one of the most common fluid-and-electrolyte abnormalities encountered in clinical practice. A normal serum potassium level is in a narrow range, from 3.5 to 5.5 meq/L. A level below 3.5 is defined as hypokalemia; the clinical manifestations include muscle weakness and cardiac arrhythmia in severe cases. In hypokalemic patients with a low total body potassium store, low serum potassium etiologies include poor potassium intake; increased potassium loss from skin, gastrointestinal tract, or kidneys; and potassium consumption due to an increased production of cells. Alkalemia or increased catecholamines or an increased insulin level from endogenic or iatrogenic exposure can lead to an intracellular shifting of potassium, causing low serum potassium while the total body potassium store remains intact. The treatment is to replenish serum potassium with a potassium supplement and correct the underlying alkalemia, excessive insulin, and catecholamine [1, 2].

Normally, potassium levels should stay the same throughout pregnancy [3]. Hypokalemia occurs in around 1% of pregnancies [2].

A cross-sectional study done in South Africa suggests pregnant women who live in a rural area, are primigravida, are younger than 25 years old, have low meat and fruit consumption, and practice geophagia are more likely to have hypokalemia during pregnancy [6]. However, there are no large, nationwide population-based studies available in the U.S. Thus, we conducted this study to analyze the prevalence and risk factors of hypokalemia in pregnancy-related hospitalizations in the U.S.

## 2. Materials and Methods

### 2.1. Study Design and Database Description

This is a retrospective cohort study of pregnancy-related hospitalizations at acute care hospitals across the U.S. Data were extracted from the Nationwide Inpatient Sample (NIS) for 2012 to 2014. The NIS was created by the Agency for Healthcare Research and Quality as a part of the Healthcare Cost and Utilization Project. In the NIS, hospitals are stratified according to ownership/control, bed size, teaching status, urban/rural location, and geographic region. A 20% random sample of all patients within each stratum is then collected, and information about patients' demographics, diagnoses, and resource utilization are entered into the database. Each discharge is then weighted to make the NIS nationally representative. The NIS is the nation's most comprehensive hospital data source enabling researchers to study healthcare delivery and patient outcomes [7]. It is a discharge-level database that contains de-identified clinical and non-clinical data elements at both the patient and hospital levels. As a result, multiple admissions for a single patient are considered separate discharges and are entered separately in the database. Patient-level data include age, sex, race, average income level of the patient's zip code of residence, principal diagnosis, and secondary diagnoses using the International Classification of Diseases, Ninth Revision, Clinical Modification (ICD 9-CM) coding system. Hospital-level data include hospital teaching status and bed size. Those elements that are directly related to an individual have been removed for confidentiality purposes. We have signed the HCUP Data Use Agreement which strictly prohibits us from making any effort to determine the identity of any person contained in the data, including patients, physicians, and other providers.

### 2.2. Study Patients

The pregnancy-related discharges included in the 2012–2014 sample were defined as any hospitalization with a pregnancy-related code (ICD-9 diagnosis codes 630 to 648) or delivery code (ICD-9 diagnosis codes 74 for Cesarean delivery and 72, 73, 75; procedure codes v27, or 650 to 659 for vaginal delivery). A post-partum admission was defined as any discharge record that included a complication of post-partum diagnosis (ICD-9 codes 670 to 677) and did not also include a delivery code. The ICD-9 code used for hypokalemia was 276.8 (hypopotassemia). Comorbidities collected were medical conditions including chronic kidney disease, sickle cell disease, systemic lupus erythematosus, congestive heart failure, obesity, coronary artery disease, Cushing's syndrome, cortico-adrenal insufficiency, and hypothyroidism. Obstetric complications collected included antepartum hemorrhage, post-partum hemorrhage, preterm labor, gestational hypertension (GH), and hyperemesis gravidarum. Comorbidities identified using the ICD-9 CM codes are presented in the supplements (available here).

### 2.3. Statistical Analysis

In our analyses, survey commands were used to account for the stratification, clustering, and weighting to produce nationally representative, unbiased results. Categorical variables were compared using a chi-squared test, and comparison of continuous values was performed using an adjusted Wald test. Univariable regression analyses were used to calculate the unadjusted odds ratio to identify risk factors of hypokalemia, while potential confounding factors (age, race, median household income, and Charlson Comorbidity Index) were included as covariates during the multivariate regression analysis and entered into a multivariable logistic regression model. All *p* values were two-sided, with 0.05 as the threshold for statistical significance. Statistical analyses were conducted using Stata, version 16.0 (StataCorp LLC, College Station, Texas).

## 3. Results

From 2012 to 2014, there were 12,431,909 pregnancy-related discharges. Of these, 85,670 (0.69%) were cases with hypokalemia. Compared with those without hypokalemia, patients with hypokalemia were younger (mean age 27.0 ± 6.2 vs. 28.1 ± 6.0, *p* < 0.001), more likely to be black, more likely to be insured by government insurance such as Medicaid, of lower household income, and with fewer comorbidities (*p* < 0.001). (Table 1).

Table 2 summarizes the medical conditions that are risk factors for pregnancy-related hypokalemia. After adjusting for confounders, medical conditions strongly associated with hypokalemia included cortico-adrenal insufficiency (adjusted odds ratio (aOR) 16.12, 95% CI 10.36–25.09, *p* < 0.001) and congestive heart failure (aOR 5.48, 95% CI 4.23–7.11, *p* < 0.001). Medical conditions significantly associated with hypokalemia included sickle cell disease (aOR 3.67, 95% CI 3.19–4.24, *p* < 0.001), systemic lupus erythematosus (aOR 1.59, 95% CI 1.29–1.97, *p* < 0.001), obesity (aOR 1.13, 95% CI 1.06–1.20, *p* < 0.001), and coronary artery diseases (aOR 2.79, 95% CI 2.00–3.89, *p* < 0.001) (Table 3).

Table 4 presents the obstetric complications found to be risk factors of pregnancy-related hypokalemia. After adjusting for confounders, complications of pregnancy significantly associated with hypokalemia were hyperemesis gravidarum (aOR 33.18, 95% CI 31.61–34.83, *p* < 0.001), post-partum hemorrhage (aOR 1.42, 95% CI 1.31–1.53, *p* < 0.001), and GH (including pre-eclampsia and eclampsia, aOR 2.03, 95% CI 1.94–2.12, *p* < 0.001) (Table 5).

## 4. Discussion

This large, nationwide population study found hypokalemia in 0.69% of pregnancy-related hospitalizations. This result is similar to the previously known incidence of 1% [2]. This supports that hypokalemia is a rare condition in pregnancy-related hospitalizations.

The result of the present study showed about 20% of pregnancy-related hospitalizations with hypokalemia also had a diagnosis of hyperemesis gravidarum, while this diagnosis occurred in less than 1% of patients without hypokalemia. This confirms that hyperemesis gravidarum is strongly associated with hypokalemia during pregnancy.

According to the present study, GH is associated with hypokalemia. This result may be explained by the suspected role of the RAAS in GH [8, 9]. Normally, volume expansion inhibits the RAAS system, leading to decreased sodium reabsorption and potassium excretion [10]. However, during pregnancy, the RAAS is altered to maintain sufficient placenta perfusion. Studies have shown that overexpression of certain angiotensin genes may be related to GH [8, 9]. The altered RAAS system is likely related to hypokalemia in patients with GH.

The present study confirms that hypokalemia during pregnancy is associated with younger age. The incidence is significantly higher at an age of less than 18 years at the time of pregnancy. The finding is also reported by Yang et al. In a cross-sectional study of 421 patients, age at pregnancy less than 25 years was found to be associated with hypokalemia [6]. The possible explanation of this finding is that the younger pregnancy age is associated with a higher incidence of hyperemesis gravidarum leading to hypokalemia [11, 12].

A median household income in the first quartile was found to be associated with hypokalemia during pregnancy. Based on the cross-sectional research done by Businge et al., pregnant patients with low fruit and meat intake tend to be associated with hypokalemia [6]. People of low socioeconomic status were reported to have a higher food insecurity and less fruit and vegetable intake due to the lack of fruit and vegetable availability [13]. This could be the possible explanation for the correlation between low socioeconomic status and hypokalemia during pregnancy. In this study, we found African American race also to be a risk factor for hypokalemia. This could be explained by the fact that African American patients are reported to have a higher risk of GH linked with altered an RAAS system, as discussed above [14].

Surprisingly, congestive heart failure, cortico-adrenal insufficiency, and sickle cell disease are associated with hypokalemia in pregnant patients. Congestive heart failure has been well known to cause hyperkalemia by decreasing the effective intravascular volume and renal perfusion [15]. However, the possible explanation of the association with hypokalemia in pregnant patients is that it could be secondary to diuretics use or an altered RAAS [8–10]. Cortisol-adrenal insufficiency is also reported to be associated with hyperkalemia by decreasing potassium excretion. However, patients with cortico-adrenal insufficiency are often treated with low doses of steroids, which can be related to hypokalemia in the setting of an altered RAAS [14]. Hyperkalemia is often induced by sickle cell crisis due to hemolysis [13]. However, in pregnant women with stable sickle cell traits or diseases, anemia has been linked to a higher percentage of gestation hypertension. The underlying mechanism could be secondary to vessel constriction caused by hypoxia. The correlation with hypokalemia could be partially explained by an altered RAAS [15, 16] There are only 140 hospitalizations with Cushing syndrome, or primary hyperaldosteronism, which was found to have no significant association with hypokalemia during pregnancy. This can indicate the low prevalence of the diseases or may be due to underdiagnosis since some of the lab results may not come back during the index hospitalization.

This is the first prevalence and risk-factor analysis in pregnancy-related hospitalizations with hypokalemia using a nationwide database covering patients in both academic and private institutions, enhancing generalizability. The main limitations of this study are the type of data available to be retrieved from a nationwide database. First, lab results are not available in the NIS. Thus, we are unable to classify the patients by severity of potassium level. Second, the available data are based on diagnostic codes from each hospitalization. Thus, we are unable to analyze the causality of each factor associated with hypokalemia or chronicity of the comorbidities. Furthermore, each observation in the dataset represents a hospitalization without patient-level information. We cannot exclude patients with multiple pregnancy admissions or access the individual medication list.

## 5. Conclusions

Based on the large, nationwide database analysis, our study suggests that the prevalence of hypokalemia during pregnancy is less than 1%. Medical conditions, including congestive heart failure, cortico-adrenal insufficiency, and sickle cell disease, are associated with hypokalemia in pregnancy-related hospitalizations. The obstetric risk factors associated with hypokalemia include post-partum hemorrhage, gestational hypertension, and hyperemesis gravidarum. More caution and monitoring are needed when taking care of patients with the aforementioned condition.

## Figures and Tables

**Table 1:** Baseline patient and hospital characteristics of pregnancy-related admissions for women with and without hypokalemia.

Demographics	Without hypokalemia	With hypokalemia	*p* ^*∗*^
No. of patients	12,346,239	85,670	
Age ± SD (y)	28.1 ± 6.0	27.0 ± 6.2	<0.001
Age group, y (%)			<0.001
1–17	2.1	3.2	
18–39	94.9	93.9	
≥40	3.0	2.9	
Race (%)			<0.001
White	52.9	43.1	
Black	15.2	31.9	
Hispanic	20.8	17.4	
Asian or Pacific Islander	5.4	2.6	
Native American	0.8	1.0	
Other	4.9	4.0	
Median household income in the patient's zip code (%)			<0.001
First quartile	28.6	40.2	
Second quartile	25.5	26.9	
Third quartile	24.5	20.3	
Fourth quartile	21.5	12.6	
Hospital bed size (%)			0.0854
Small	13.5	12.7	
Medium	29.4	28.9	
Large	57.1	58.4	
Primary payer (%)			<0.001
Government insured	46.5	62.4	
Private	50.7	31.9	
Self-pay	2.9	5.7	
Teaching hospital (%)	55.8	59.5	<0.001
Charlson Comorbidity Index score (%)			<0.001
0	94.2	80.7	
≥1	5.8	19.3	

All *p* values were two sided, with 0.05 as the threshold for statistical significance.

**Table 2:** Medical conditions of pregnancy-related admissions for women with and without hypokalemia.

Medical conditions	Without hypokalemia	With hypokalemia	*p* ^*∗*^
Chronic kidney disease (%)	0.07	0.63	<0.001
Sickle cell disease (%)	0.26	1.35	<0.001
Systemic lupus erythematosus (%)	0.15	0.60	<0.001
Congestive heart failure (%)	0.03	0.73	<0.001
Obesity (%)	6.3	8.88	<0.001
Coronary artery disease (%)	0.03	0.37	<0.001
Cushing's syndrome (%)	0.002	0.02	<0.001
Cortico-adrenal insufficiency (%)	0.01	0.21	<0.001
Hypothyroidism (%)	2.8	2.9	0.46

All *p* values were two sided, with 0.05 as the threshold for statistical significance.

**Table 3:** Medical conditions and the risk of pregnancy-related hypokalemia.

Medical condition	Univariate analysis				Multivariate analysis			
	OR	*p* value	95% CI lower	95% CI upper	OR	*p* value	95% CI lower	95% CI upper
Chronic kidney disease	8.91	<0.001	7.23	11.00	1.14	0.334	0.88	1.47
Sickle cell disease	5.28	<0.001	4.59	6.07	3.67	<0.001	3.19	4.24
Systemic lupus erythematosus	4.01	<0.001	3.28	4.88	1.59	<0.001	1.29	1.97
Congestive heart failure	27.70	<0.001	22.59	33.96	5.48	<0.001	4.23	7.11
Obesity	1.44	<0.001	1.36	1.53	1.13	<0.001	1.06	1.20
Coronary artery disease	9.98	<0.001	7.66	12.99	2.79	<0.001	2.00	3.89
Cushing's syndrome	13.73	<0.001	5.10	36.97	4.30	0.064	0.92	20.03
Cortico-adrenal insufficiency	18.40	<0.001	12.77	26.25	16.12	<0.001	10.36	25.09
Hypothyroidism	1.04	0.46	0.94	1.14				

**Table 4:** Obstetric complications of pregnancy-related admissions for women with and without hypokalemia.

Obstetric complications	Without hypokalemia	With hypokalemia	*p* ^*∗*^
Antepartum hemorrhage (%)	1.78	2.02	0.0194
Post-partum hemorrhage (%)	3.46	4.71	<0.001
Preterm labor (%)	7.48	7.44	0.8526
Gestational hypertension (%)	10.68	22.10	<0.001
Hyperemesis gravidarum (%)	0.71	22.44	<0.001

All *p* values were two sided, with 0.05 as the threshold for statistical significance

**Table 5:** Obstetric complications and the risk of pregnancy-related hypokalemia.

Pregnancy or delivery complication	Univariate analysis				Multivariate analysis			
	OR	*p* value	95% CI lower	95% CI upper	OR	*p* value	95% CI lower	95% CI upper
Antepartum hemorrhage	1.14	0.019	1.02	1.27	1.09	0.151	0.97	1.22
Post-partum hemorrhage	1.38	<0.001	1.28	1.49	1.42	<0.001	1.31	1.53
Preterm labor	0.99	0.853	0.93	1.06				
Gestational hypertension	2.37	<0.001	2.27	2.48	2.03	<0.001	1.94	2.12
Hyperemesis gravidarum	40.28	<0.001	38.54	42.11	33.18	<0.001	31.61	34.83

## Data Availability

Publicly available datasets were analyzed in this study. These data can be found at https://www.hcup-us.ahrq.gov/db/nation/nis/nisdbdocumentation.jsp.

## Supplementary Materials

Supplementary Materials Supplement Table 1: International Classification of Diseases (ICD-9) code for medical conditions. Supplement Table 2: International Classification of Diseases (ICD-9) code for obstetric complications.
