# Supplementary material for: The Prevalence and Risk Factors of Hypokalemia in Pregnancy-Related Hospitalizations: A Nationwide Population Study
Source: Int J Nephrol. 2021 Jun 28;2021:9922245. doi: 10.1155/2021/9922245 (PMC8261188; doi:10.1155/2021/9922245)
Supplement: Supplementary Materials — Supplement Table 1: International Classification of Diseases (ICD-9) code for medical conditions. Supplement Table 2: International Classification of Diseases (ICD-9) code for obstetric complications. [file 9922245.f1.docx]

**Supplement Table 1. International Classification of Diseases (ICD-9) code for medical conditions**

| Chronic kidney disease | 585.1 Chronic kidney disease, Stage I  585.2 Chronic kidney disease, Stage II (mild)  585.3 Chronic kidney disease, Stage III (moderate)  585.4 Chronic kidney disease, Stage IV (severe)  585.5 Chronic kidney disease, Stage V  585.9 Chronic kidney disease, unspecified |
| --- | --- |
| Sickle cell disease | 282.5 Sickle-cell trait  282.6 Sickle-cell disease |
| Systemic lupus erythematosus | 710.0 Systemic lupus erythematosus |
| Congestive heart failure | 428.22 Chronic systolic heart failure  428.23 Acute on chronic systolic heart failure  428.32 Chronic diastolic heart failure  428.33 Acute on chronic diastolic heart failure  428.42 Chronic combined systolic and diastolic heart failure  428.43 Acute on chronic combined systolic and diastolic heart failure |
| Obesity | 278.0 Overweight and obesity  649.1 Obesity complicating pregnancy, childbirth, or the puerperium  V85.3 Body mass index between 30-39, adult  V85.4 Body mass index 40 and over, adult |
| Coronary artery disease | 411 Other acute and subacute forms of ischemic heart disease  412 Old myocardial infarctions  413 Angina pectoris  414 Other forms of chronic ischemic heart disease |
| Cushing’s syndrome | 255.0 Cushing's syndrome |
| Cortico-adrenal insufficiency | 255.41 Glucocorticoid deficiency  255.42 Mineralocorticoid deficiency |
| Hypothyroidism | 244 Acquired hypothyroidism |

**Supplement Table 2. International Classification of Diseases (ICD-9) code for obstetric complications**

| Antepartum Hemorrhage | 640.9 Unspecified hemorrhage in early pregnancy  641.1 Hemorrhage from placenta previa  641.2 Premature separation of placenta  641.3 Antepartum hemorrhage associated with coagulation defects  641.8 Other antepartum hemorrhage  641.9 Unspecified antepartum hemorrhage |
| --- | --- |
| Postpartum Hemorrhage | 666.0 Third-stage postpartum hemorrhage  666.1 Other immediate postpartum hemorrhage  666.2 Delayed and secondary postpartum hemorrhage  666.3 Postpartum coagulation defects  667 Retained placenta or membranes without hemorrhage  669.1 Obstetric shock |
| Preterm Labor | 644 Early or threatened labor |
| Gestational Hypertension | 642 Hypertension complicating pregnancy childbirth and the puerperium |
| Hyperemesis Gravidarum | 643 Excessive vomiting in pregnancy |
